# A Novel *in situ* Approach to Studying Detrusor Smooth Muscle Cells in Mice

**DOI:** 10.1038/s41598-020-59337-0

**Published:** 2020-02-14

**Authors:** Tamara Serdinšek, Saša Lipovšek, Gerd Leitinger, Igor But, Andraž Stožer, Jurij Dolenšek

**Affiliations:** 1 Department of General Gynaecology and Urogynaecology, Clinic for Gynaecology and Perinatology, University Medical Centre Maribor, Ljubljanska 5, 2000 Maribor, Slovenia; 2 Faculty of Medicine, University of Maribor, Taborska ulica 8, 2000 Maribor, Slovenia; 3 Faculty of Natural Sciences and Mathematics, University of Maribor, Koroška cesta 160, 2000 Maribor, Slovenia; 4 Faculty of Chemistry and Chemical Engineering, Smetanova ulica 17, University of Maribor, 2000 Maribor, Slovenia; 5 Gottfried Schatz Research Center, Division of Cell Biology, Histology and Embryology, Medical University of Graz, Neue Stiftingtalstrasse 6, 8010 Graz, Austria

**Keywords:** Bladder, Fluorescence imaging

## Abstract

The aim of our study was to develop a novel approach to investigating mouse detrusor smooth muscle cell (SMC) physiological activity, utilizing an acute tissue dissection technique and confocal calcium imaging. The bladder of a sacrificed adult female NMRI mouse was dissected. We used light and transmission electron microscopy to assess morphology of SMCs within the tissue. Calcium imaging in individual SMCs was performed using confocal microscopy during stimulation with increasing concentrations of carbamylcholine (CCh). SMCs were identified according to their morphology and calcium activity. We determined several parameters describing the SMC responses: delays to response, recruitment, relative activity, and contraction of the tissue. CCh stimulation revealed three different SMC phenotypes: spontaneously active SMCs with and without CCh-enhanced activity and SMCs with CCh-induced activity only. SMCs were recruited into an active state in response to CCh-stimulation within a narrow range (1–25 µM); causing activation of virtually all SMCs. Maximum calcium activity of SMCs was at about 25 µM, which coincided with a visible tissue contraction. Finally, we observed shorter time lags before response onsets with higher CCh concentrations. In conclusion, our novel *in situ* approach proved to be a robust and reproducible method to study detrusor SMC morphology and physiology.

## Introduction

Investigating detrusor muscle physiology and responses to different stimuli is a challenging task because of its complex physiology, occurrence of functional and morphological changes under pathophysiological conditions, and anatomic and physiologic differences in detrusors of different species^1,2^. To date, several different *in vitro* techniques for investigating urinary bladder physiology have been described, each with inherent drawbacks. They can be broadly divided into (i) techniques utilizing freshly isolated smooth muscle cells (SMCs), (ii) cell culture techniques, and (iii) tissue dissection techniques. Cell culture techniques can be further divided into explant and enzymatic methods^3^.

Tissue dissection techniques are especially useful in detrusor contractility investigations. In these methods, the urinary bladder is dissected and the urothelial and the serous layer are usually removed^4,5^, since responses to pharmacological stimuli can be altered in the presence of mucosa^6^. Bladder tissue of different species has been investigated using this approach^7,8^.

Several tissue dissection techniques have been described in the literature and they share the following common steps: (i) urinary bladder dissection, (ii) removal of the mucosal and serous layer, (iii) cutting of the bladder into smaller pieces, and (iv) transfer to a dissection solution with or without an equilibration period in a calcium-free solution. The purpose of the equilibration period is to stabilize spontaneous detrusor contractions. Its duration varies according to different authors and can be as long as two hours. Before the beginning of the experiment, calcium-free dissection solution is replaced with a solution that contains calcium^4–7^.

For example, a widely used technique described by Kullman *et al*. in 2014 consists of removing the urinary bladder from an anesthetized animal, putting it in Krebs solution and cutting it into strips. Strips are then placed into a chamber filled with a warm Krebs solution, attached to isometric tension transducer on one end and to a fixed rod on the other end. The method requires 1–2 hours of tissue equilibration with consistent washing using a warm aerated Krebs solution to achieve a stable baseline tension. Strips can be stimulated with either pharmacological or neural stimuli^6^. Bladder strips have been employed over the years to evaluate changes in myogenic and neuronal factors that affect bladder function^6,9^, to compare differences between tissues of different species, organs, and for evaluation of relationships between drug structure and tissue activity, and relevance of specific bladder components (e.g., bladder mucosa)^6^.

Wuest *et al*. described a similar detrusor muscle strip method in which strips were mounted in Tyrode’s solution after dissection and mucosa removal. Tension generated by the strips was measured with an isometric force transducer. After the adjustment of the resting load during the 60-minute equilibration period, strips were exposed to CCh twice. After an additional equilibration period of 20 minutes, strips were stimulated with CCh or electrical field^7^. This particular method has been used several times in investigation of bladder physiology and determining the effect of different drugs on the detrusor contractility^7,10–12^.

Although widely used, there are still several limitations of these methods. First, different methods can achieve highly variable numbers of isolated SMCs, purity of the isolate, and percentage of relaxed SMCs, which can significantly influence their efficacy. Both cell and tissue dissection techniques can alter the function of detrusor SMCs, which can affect the investigation results; for example, there is a well-known phenomenon of phenotypic plasticity in cell cultures techniques. Moreover, tissue dissection techniques have the disadvantage of a relatively long equilibration period and limited tissue viability, usually for a couple of hours. These drawbacks limit our understanding of bladder SMC (patho)physiology and therefore necessitate an alternative, more *in vivo* approach. To this aim, we developed a novel methodological approach to study detrusor physiology in mice and investigated the possibilities of its application. Our goal was to establish a method that would simultaneously allow investigation of the tissue slice as a whole and at the level of individual SMCs, and also be simple, fast, reproducible, and cost-effective at the same time.

## Study Design, Materials, and Methods

Adult female NMRI mice were used in the experiments. Prior to the study, we obtained an approval from the National medical ethics committee (approval number KME 98/05/16) and from The Administration of the Republic of Slovenia for food safety, veterinary, and plant protection (approval number U34401-13/2016/2). All methods were carried out in accordance with the guidelines from local authorities, i.e. The Administration of the Republic of Slovenia for food safety, veterinary, and plant protection.

### Tissue dissection

**Figure 1:**
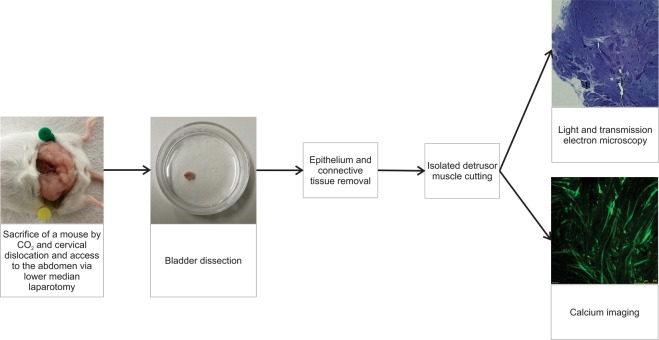
Flowchart of the acute mouse detrusor muscle dissection technique. Flowchart was constructed using CorelDraw Graphics Suite X7 (https://www.coreldraw.com/en/).

For tissue dissection, adult female NMRI mice were sacrificed by utilizing CO_2_ and cervical dislocation. Immediately thereafter, abdomen was accessed via lower median laparotomy and the bladder was dissected carefully. The dissected bladder was immediately transferred into an ice-cold calcium-free Krebs solution. Subsequently, connective tissue and urothelium were gently removed from detrusor muscle under a light stereomicroscope (Nikon SMZ 745). The isolated detrusor muscle was cut into three to four 2–4 mm^2^ pieces using sharp scissors and transferred into a fresh ice-cold calcium-free Krebs solution, where it was incubated for 15–20 minutes. Next, tissue slices were loaded with the membrane–permeable calcium reporter dye Fluo-4-AM (ThermoFisher, 5 µM), with dimethyl sulfoxide (DMSO, 0.1% v/v) and Pluronic (Invitrogen, 0.4% v/v) in a HEPES-buffered solution (HBS) for 30–40 minutes at room temperature on a shaker at 50 strokes per minute. Dissected tissue was then transferred into an ice-cold HBS and kept on ice until calcium imaging (Fig. 1)^13^.

### Light microscopy and transmission electron microscopy

We fixed small pieces of the urinary bladder in 2.45% glutaraldehyde and 2.45% paraformaldehyde in a 0.1 M sodium cacodylate buffer (pH = 7.4) at room temperature for 4 hours and at 4 °C for 16 hours. We washed the tissue in a 0.1 M sodium cacodylate buffer (pH = 7.4) at room temperature for 3 hours and post-fixed with 2% OsO_4_ at room temperature for 2 hours. Afterwards the tissue was dehydrated in a graded series of ethanol (50%, 70%, 90%, 96%, and 100%, each for 30 min at room temperature) and embedded in the TAAB epoxy resin (Agar Scientific)^14^. For light microscopy, we stained semi-thin sections (500 nm) of the tissue with 0.5% toluidine blue in an aqueous solution and analysed by a Nikon Eclipse E800 light microscope equipped with a Nikon DN100 camera. For transmission electron microscopy, we prepared ultra-thin sections (75 nm) and transferred them onto copper grids. Additionally, they were stained with uranyl acetate and lead citrate and analysed by a Zeiss EM 902 transmission electron microscope.

### Confocal calcium imaging

Calcium imaging was performed immediately following dye incubation using the inverted confocal microscope Leica TCS SP5 II. Calcium dye was excited at 488 nm and emitted fluorescence was detected with a HyD detector (range 500–600 nm), pixel size 512 × 512 at 1 Hz. For calcium imaging, individual slices were transferred into the recording chamber and perfused with HBS at 37 °C. To investigate responses of the detrusor muscle to pharmacological stimuli, tissue slices were stimulated with increasing concentrations of the cholinomimetic drug carbamylcholine (carbachol, CCh) in HBS according to the following stimulation protocol: HBS only, 1 µM, 10 µM, 25 µM, 50 µM, and 100 µM CCh, and then wash-out using HBS only. Each step of the protocol lasted for 300 seconds^13^.

### Solutions and drugs

The calcium-free Krebs solution contained 112 mM NaCl, 4.7 mM KCl, 2.2 mM CaCl_2_, 1.2 mM MgCl_2_, 25 mM NaHCO_3_, 1.2 mM KH_2_PO_4_, and 14 mM glucose, and was continuously gassed with carbogen (95% O_2_, 5% CO_2_). HBS contained 137 mM NaCl, 5.9 mM KCl, 2.2 mM CaCl_2_, 1.2 mM MgCl_2_, 14 mM glucose, and 10 mM HEPES. The pH was adjusted to 7.4 with NaOH. CCh for SMC stimulation was prepared in HBS in different concentrations according to the abovementioned protocol. All chemicals were from Sigma Aldrich, unless stated otherwise.

### Data analysis

Obtained time series were analysed off-line using custom Matlab software and MS Excel. SMC were identified according to their morphology and ROIs were handpicked for further analysis. If needed, ROI positions were corrected to follow SMC physical displacement during tissue contraction. Time series data were fitted using a combination of linear and exponential curve to account for photo-bleaching. Following parameters were analysed: (i) presence of SMC activity, (ii) onset of SMC activity, (iii) relative activity of SMCs, and (iv) contraction of the tissue. Based on these parameters, phenotypes of SMC responses were identified, cumulative percentage of activated SMC during each step of stimulation was calculated, and threshold concentration for tissue slice contraction was determined. To compare data on percentage of active SMCs, onset of SMC activity and relative activity of SMCs during stimulation with different concentration of CCh, ANOVA on ranks was performed using SigmaPlot software. Statistical significance was set at p < 0.05. For dose-response and time to response curves, data were fitted to a sigmoidal function using the following equation: f(x) = a/(1 + exp (−(x − x°)/b)). To calculate relative activity of individual SMCs, time series were adjusted to fit the 2^8^ range, displayed as one pixel row per SMC, and further used for statistical analysis. A smoothing spline function was used to create fit for the SMC relative activity curve.

To quantify tissue contraction, maximum tissue displacement after CCh stimulation was measured in individual preparations and interpreted as evidence for a contractile tissue response. To this aim, 10–150 frames were averaged before and after CCh-induced contraction and displayed in false green and red colour, respectively. An overlay of the two allowed for exact tissue displacement determination using Fiji software. The maximum displacement per tissue preparation was used for statistical analysis.

All images and plots were prepared using Matlab 2013b (https://www.mathworks.com/products/matlab.html) and CorelDraw Graphics Suite X7 (https://www.coreldraw.com/en/).

## Results

Our acute tissue dissection technique in combination with confocal calcium imaging proved to be a very efficient method. Success rate for tissue slice preparation was 100% and for obtaining responsive SMCs during calcium imaging 73.6% (N = 5 animals). The success rate for successful imaging was further improved by prolonging the calcium dye loading time from 30 minutes to 40 minutes. On average, three tissue detrusor tissue pieces having convenient surface for calcium imaging (approximately 2–4 mm^2^) were obtained from single urinary bladder.

**Figure 2:**
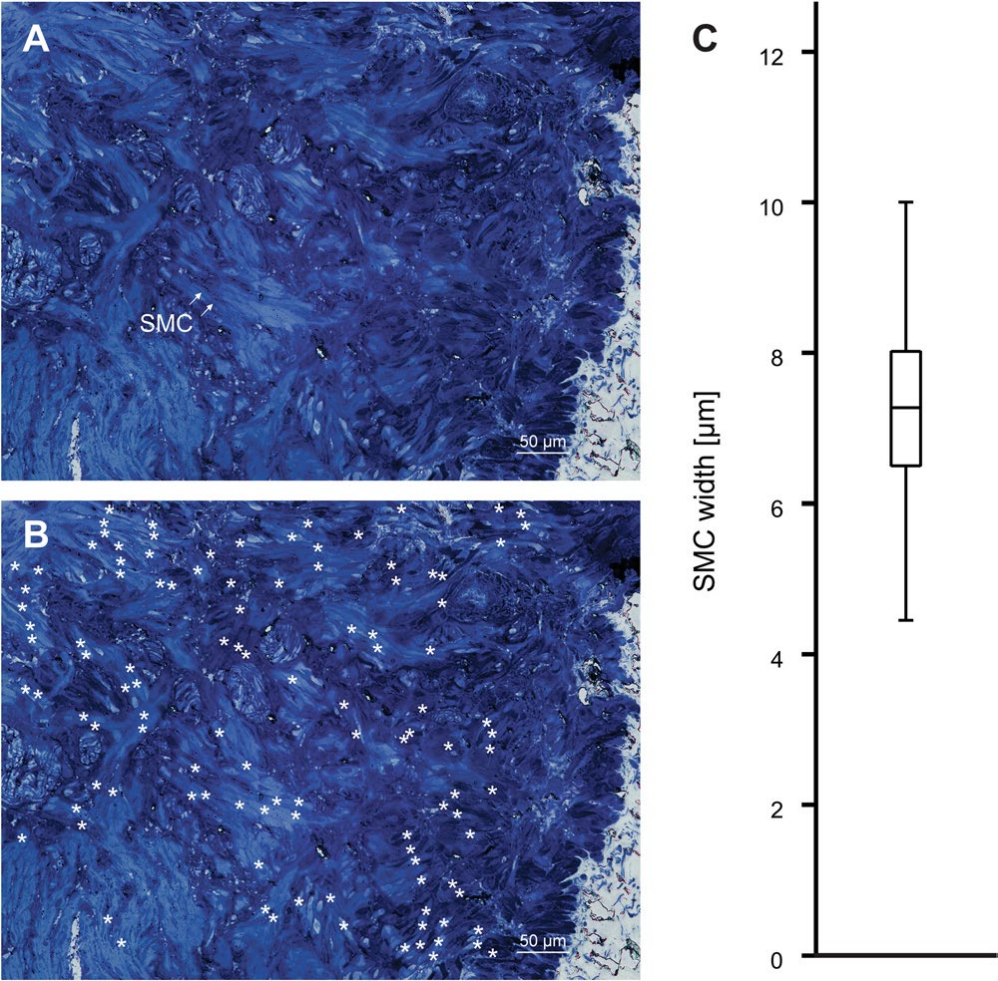
Light microscopy of a mouse detrusor muscle stained with 0.5% toluidine blue. (**A**) A smooth muscle cell (SMC) was visible as a spindle-shaped cell oriented in different directions. Arrows indicate one SMC out of many visible in the micrograph. (**B**) Individual SMCs are marked with asterisk on the same micrograph as in (**A**). Note that more than 100 SMCs were visible within a single preparation. (**C**) The width of detrusor SMCs measured on the micrographs (N = 46 cells measured in one tissue section).

**Figure 3:**
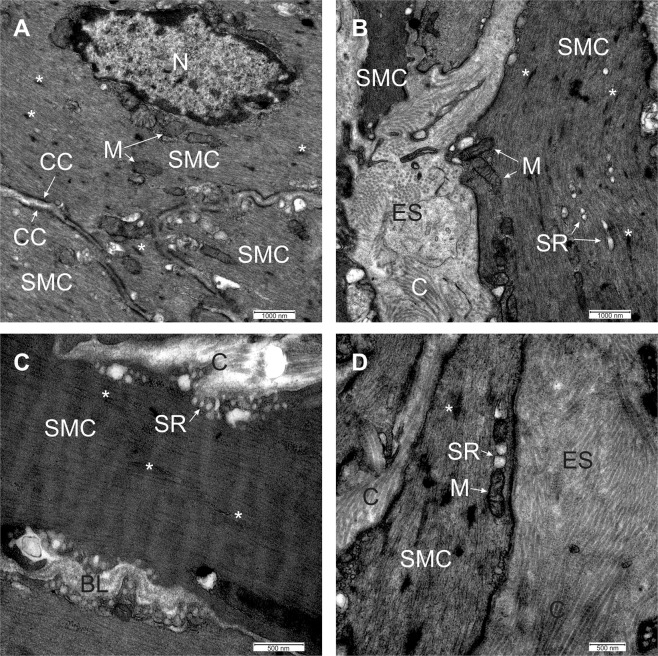
Transmission electron microscopy of a mouse detrusor muscle. (**A**) The nucleus (N) of the smooth muscle cell (SMC) is oval and located centrally. The sarcoplasm is filled with filaments, some of them are forming electron-dense regions (asterisks). Many mitochondria (M) are present in the sarcoplasm, especially in the vicinity of the nucleus, and cell contacts (CC) can clearly be discerned. (**B**) In the smooth muscle cell (SMC) filaments (asterisks), mitochondria (M), and some cisternae of the sarcoplasmic reticulum (SR) are present. Numerous collagen fibrils (**C**) are in the extracellular space (ES). (**C**) The cisternae of the sarcoplasmic reticulum (SR) are located close to the sarcolemma. Around the SMC, the basal lamina (BL) is present, and electron-dense regions in the sarcoplasm (asterisks) are formed by the filaments. (**D**) Mitochondria (M) are found in the close vicinity of the smooth reticulum (SR). Electron-dense regions in the sarcoplasm (asterisk) are formed by the filaments, and collagen fibrils (**C**) are clearly visible in the extracellular space (ES).

First, to characterize morphology of SMCs obtained with our approach, we resorted to light and transmission electron microscopy. Bladder SMCs have a fusiform shape when observed under light microscopy (Fig. 2A,B). The median width of SMCs in light microscope sections measured at their widest point was 7.3 µm (Q_1_ = 6.5 µm and Q_3_ = 8 µm, N = 46 cells) (Fig. 2C). Because SMCs in tissue sections were transected at different levels during preparation for light microscopy, their length could not be reliably measured, however maximal lengths that we observed were up to 400 µm. They were grouped in branching bundles. These bundles were not oriented strictly parallel and ordered, but were forming a very complex system (Fig. 2A). We next looked at the ultrastructure of the SMCs. The nuclei of SMCs were oval or irregularly shaped and located centrally (Fig. 3A). The SMCs were also characterized by numerous electron-lucent and electron-dense filaments (Fig. 3A–D), filling most of the sarcoplasm. In each SMC, electron-dense filaments seemed to be grouped in thicker formations having an electron-dense appearance (see asterisks in Fig. 3A–D). Some cisternae of the sarcoplasmic reticulum were seen in the central part of the cells (Fig. 3B) or in close vicinity of the cell membrane (Fig. 3C). Oval mitochondria were located in different parts of the cell, but more frequently close to the nucleus (Fig. 3A) or close to the sarcoplasmic reticulum (Fig. 3D). Between neighbouring SMCs, electron-dense cell contacts could be observed (Fig. 3A). Individual SMCs were surrounded by electron-lucent basal lamina (Fig. 3C). The extracellular space was electron-lucent, containing numerous fibrils, probably collagen (Fig. 3B–D).

In addition to SMCs, interstitial cells of Cajal (ICC) were also identified in TEM sections. ICCs are bipolar cells forming some lateral cytoplasmic extensions. Where ICCs are oriented parallel to each other, some extensions reach toward neighbouring ICCs (Supplemental Fig. 1A), making close contacts with each other. Additionally, the extensions of the ICCs make contacts with surrounding SMCs (Supplemental Fig. 1B). Supplemental Fig. 1B also shows one part of an ICC surrounded by SMCs in close contact (up to 90 nm), which could be important for communication between the cells. ICCs are characterized by rough endoplasmic reticulum (Supplemental Fig. 1A), electron-lucent caveolae (Supplemental Fig. 1A,B) and electron-lucent intracellular vesicles (Supplemental Fig. 1A inset and B). This is in stark contrast to SMCs, which contain many electron dense filaments and individual bands of electron dense cell membrane. These structures were not observed in ICCs.

**Figure 4:**
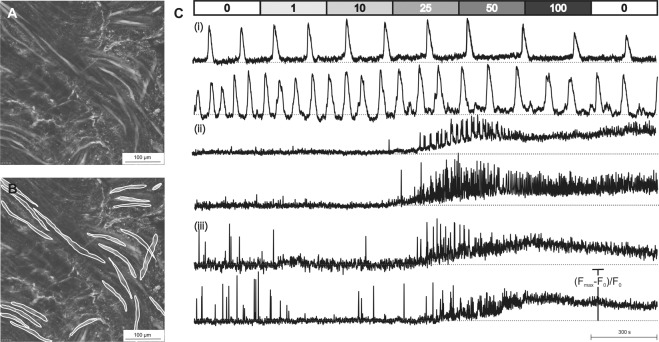
Carbamylcholine (CCh) stimulation revealed three phenotypes of SMC calcium dynamics. (**A**) Detrusor muscle tissue loaded with Fluo-4 AM. (**B**) Same preparation as in A, depicting the outlines of individual SMCs. (**C**) Spontaneously active SMCs that failed to respond to CCh (phenotype (i)), SMCs with CCh-induced oscillatory activity, without previous spontaneous activity (phenotype (ii)), and spontaneously active SMCs with CCh-enhanced oscillatory activity (phenotype (iii)).

After testing several calcium-sensitive dyes and different loading protocols, we optimized the dye-loading protocol as described in detail in Methods. In short, the optimal loading time was 30–40 minutes at room temperature using 5 µM of Fluo-4-AM with added DMSO and Pluronic in HBS. Besides Fluo-4-AM, two additional calcium dyes with different excitation and emission spectra and with different K_d_ values for calcium were tested: OGB-1-AM and Rhod-2-AM. In OGB-1-AM stained tissue, the SMCs were clearly visible and their responses to stimulation by CCh closely resembled the ones obtained by Fluo-4-AM. Rhod-2-AM loading allowed for SMC visualization; however, no detectable calcium activity could be detected, in spite of visible tissue contraction that served as a positive control confirming viability of tissue (Supplemental Fig. 2). SMCs loaded with Fluo-4-AM were easily identified, as they appeared as elongated spindle-like structures (Fig. 4A,B). Fluo-4 loaded into the SMCs to different levels within the same preparation. Importantly, the level of dye loading did not affect SMC responses (Supplemental Fig. 3). Interestingly, the slight differences in loading or basal calcium levels in different cells actually helped identify individual cells from one another, especially in regions where they were more closely packed together, similarly to tissue preparations from other organs^15–19^.

To characterize physiological SMC calcium responses, we used stimulation with CCh. CCh is a cholinomimetic drug that binds and activates acetylcholine receptors and has been used as a stimulating agent for detrusor SMCs previously^7,9,10,12^. After CCh stimulation, a typical response consisted of a transient increase in the intracellular calcium concentration with superimposed increase in oscillatory activity (Fig. 4 and Supplemental Video 1). In addition to SMC activity, ICC-like activity was detected from spindle-shaped cells of much smaller size (Supplementary Fig. 1C–E). Their activity consisted of highly regular calcium oscillations that were not synchronized with other ICCs or with SMCs. Because we relied on the size, shape, and position of ICC with regard to SMCs to identify them, it was sometimes not possible to reliably differentiate between ICC and transversely-cut SMCs with spontaneous activity, especially since detrusor SMCs have also been found to be able to generate spontaneous action potentials^20^. Because the main focus of this paper were SMCs, and more importantly, since the ICC-like activity was not correlated with SMC activity, thus indicating weak or no coupling of the two, we did not analyse the ICCs further. In future studies, their responses to other concentrations of CCh and other stimuli should be assessed, together with their immunocytochemical identification after the functional studies.

In an attempt to further functionally characterize SMCs, classification in phenotypes was done based upon stimulation with increasing concentrations of CCh. While some SMCs exerted spontaneous activity prior to stimulation, others responded only after stimulation with CCh. Based on these observations, we identified three different phenotypes of SMC responses (Fig. 4C):

i. SMCs with spontaneous activity in the form of calcium oscillations prior to CCh stimulation that remained unaffected by CCh stimulation (38.2% of all cells, N = 58),
ii. SMCs without spontaneous activity that responded to stimulation with CCh with a transient increase in intracellular calcium concentration with superimposed oscillatory activity (49.3% of all cells, N = 75),
iii. SMCs with spontaneous activity prior to stimulation and transient increase in intracellular calcium concentration with superimposed increased oscillatory activity after CCh stimulation (12.5% of all cells, N = 19).

**Figure 5:**
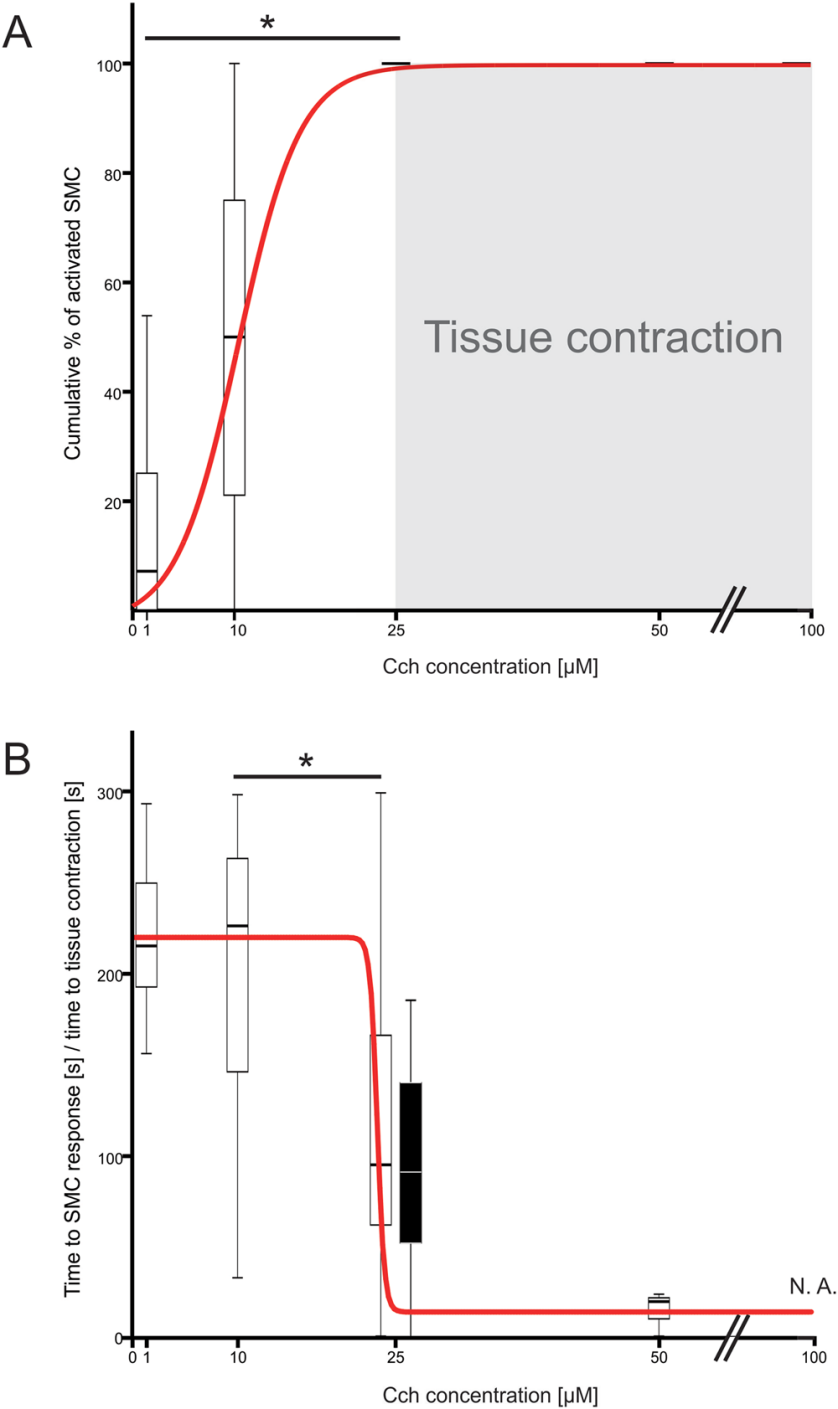
Dose-dependence of SMC responses to CCh in the acute tissue preparation. (**A**) Cumulative percentage of activated SMCs during stepwise stimulation with increasing concentrations of CCh. The grey area depicts visible tissue contraction as a function of CCh concentration. (**B**) Time lags after stimulation onset for individual SMC responses to CCh (white boxplots and red curve) and for visible tissue response (black boxplot). Legend: *p < 0.05.

**Figure 6:**
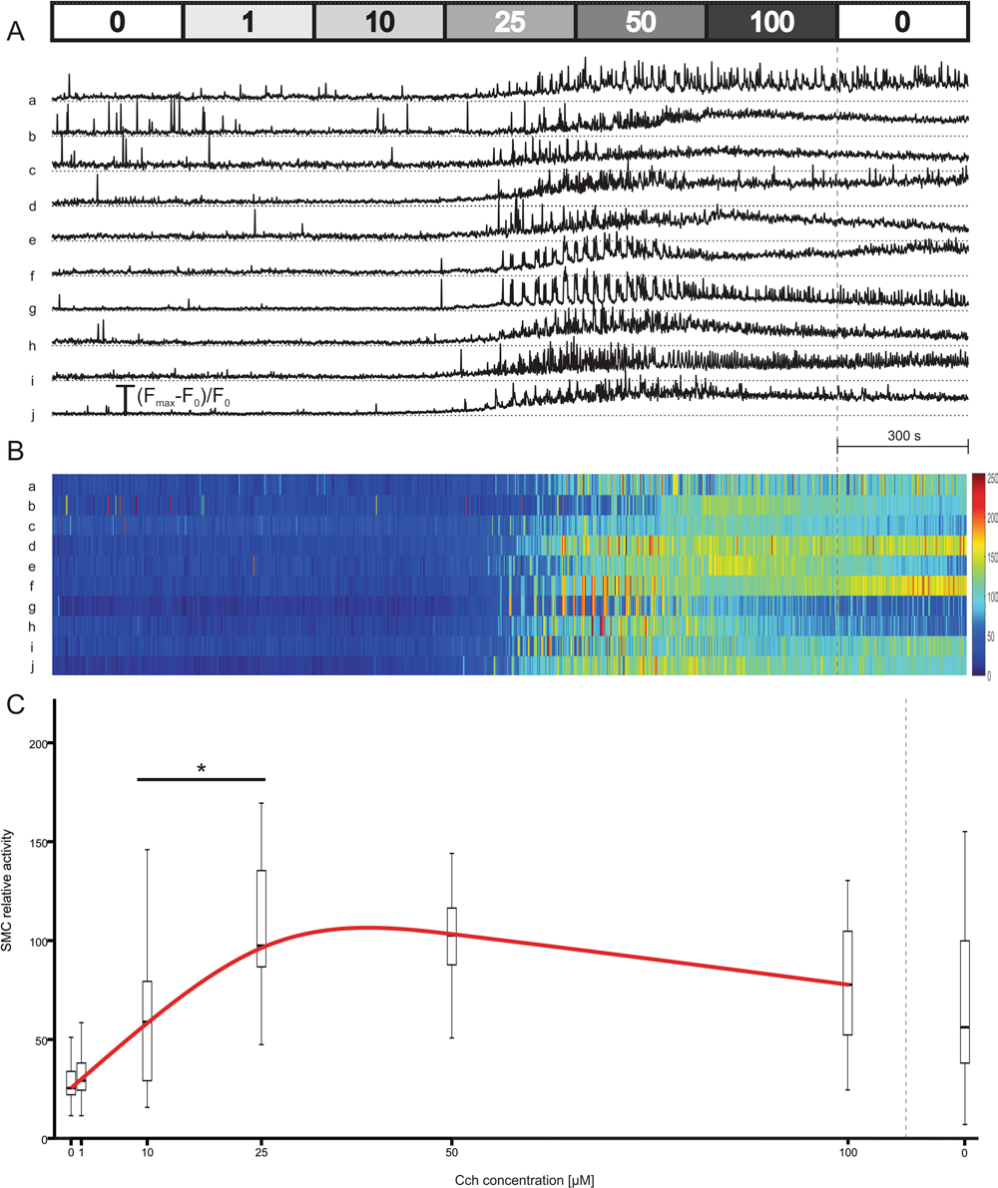
Individual SMC responses to CCh. (**A**) Individual SMC responses to CCh from one tissue preparation. Shown are only the phenotypes (ii) and (iii). Individual SMCs are marked with letters a-j. (**B**) SMC activity from (**A**) presented with false colours, letters a-j correspond to SMCs in (**A**). (**C**) Quantification of SMC relative activity as a function of CCh. Pooled data from 32 SMCs. Legend: *p < 0.05.

**Figure 7:**
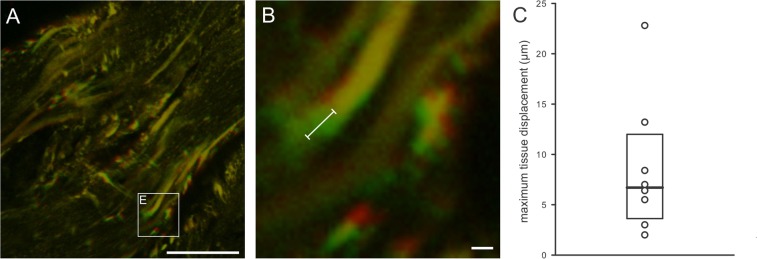
Tissue contraction following CCh stimulation. (**A**) Exemplary Fluo-4 loaded detrusor tissue before and after CCh stimulation. Shown is a merged figure of cell location before (green) and after (red) CCh-induced contraction. Yellow pixels indicate an unchanged location during contraction. Indicated is the area that is magnified in (**B**). Scale bar is 100 µm. (**B**) Area indicated in A depicting displacement of individual cells from the same preparation, false colour interpretation is the same as in A. The white line indicates maximum displacement during contraction that was detected in this preparation. Scale bar is 10 µm. (**C**) Quantification of the maximum tissue displacement following stimulation with CCh, which was used as a surrogate for measurement of the tissue contraction. Data pooled from 8 tissue preparations from five mice. Circles indicate individual values, median = 6.7 µm, Q1 = 4.3 µm, and Q3 = 10.8 µm.

We analysed SMC responses during the staircase protocol into more detail. First, we characterized recruitment of SMCs as the stimulus concentration increased. As expected, the percentage of activated SMCs (for this purpose, we merged phenotypes (ii) and (iii)) increased with increasing concentrations of CCh. Surprisingly, the dose-dependency curve was relatively steep as increasing CCh from 1 µM to 25 µM CCh caused the activation of virtually all (96.8%, N = 91) SMCs (Fig. 5A). Second, we were interested in time lags between stimulation and the SMC response. The average time from the beginning of the stimulation to the CCh-induced activity was progressively shorter in higher concentrations of CCh with statistically significant difference between 10 µM and 25 µM CCh. The median time to response for SMC with CCh-induced activity at 1 µM was 215 seconds (Q_1_ = 193, Q_3_ = 251), at 10 µM 226 seconds (Q_1_ = 146, Q_3_ = 266), at 25 µM 95 seconds (Q_1_ = 63, Q_3_ = 167) and at 50 µM 20 seconds (Q_1_ = 1, Q_3_ = N.A.) (Fig. 5B). Importantly, not all intracellular calcium increases were accompanied by a mechanical contraction. The minimum concentration that evoked a visible contraction was 25 µM CCh in all tissue slices tested, and the median time until visible tissue slice contraction was 92 seconds (Q_1_ = 35.5, Q_3_ = 152.0) from the beginning of the stimulation at this concentration (Fig. 5B). Finally, we analysed relative activity of SMCs during the staircase CCh protocol. To this aim, we compared relative change in fluorescence in SMCs subjected to the same staircase protocol. CCh progressively activated SMCs with statistically significant increase in relative SMC activity between 10 µM and 25 µM CCh. The peak of cell activity was between 25 and 50 µM CCh (Fig. 6). The median value of maximum tissue displacement after CCh stimulation was 6.7 µm (Q_1_ = 4.3 µm and Q_3_ = 10.8 µm) (Fig. 7).

## Discussion

We developed a novel approach to study detrusor muscle physiology utilizing a combination of acute detrusor tissue preparation and confocal calcium imaging. Tissue preparation methodology is described in detail in the methods section; its main cornerstones were: (i) acute tissue isolation, (ii) removal of the urothelial layer to limit the tissue to SMCs only, and (iii) easy access to obtaining tissue for further imaging on the confocal microscope. The technique proved to be relatively fast. The time from animal sacrifice to calcium imaging was about 90 minutes. Importantly, this methodology enabled us to use the tissue over long periods of time, as we regularly obtained successful, repetitive, and comparable responses of SMCs even after four to five hours after tissue preparation, provided we kept the tissue in ice-cold HBS.

Light and transmission electron microscopy confirmed the presence of SMCs in the dissected tissue, with observed shape and size of individual SMCs being similar to observations in other studies^21^. SMCs were grouped in branching bundles with fibrils that probably represent collagen placed in-between them, which is comparable to microscopic findings of Hornsby *et al*.^22^. Some studies also described the presence of elastin fibres in addition to collagen^23^. Different data can be found in the literature regarding the orientation of murine detrusor smooth muscle fibres. Shchueth *et al*. described both longitudinally and circularly oriented smooth muscle fibres in murine detrusor, although the organization of these fibres seemed to be age-related, with older mice having more intermingled collagen and smooth muscle layers^24^. On the other hand, our findings are more consistent with some other authors that describe murine detrusor muscle as composed of irregularly oriented muscle fibres^25^. Besides these structures, interstitial cells can also be identified in the outer part of the detrusor muscle layer using immunofluorescence^26^.

*In vivo* detrusor muscle responses in mammals can be initiated either by stimulation of parasympathetic nerves, by field stimulation of intrinsic excitatory nerves or SMCs, or by a direct application of acetylcholine, CCh or other agonists to the muscle strips^7,27^. In cultured SMC studies, histamine also proved to be a very effective mediator of calcium release^27^. In our study, we chose stimulation with the cholinomimetic CCh, which previously induced a strong concentration-dependent contraction in both murine and pig strips^6^. In detrusor muscle, muscarinic receptors M2 are predominant, but M3 type plays a key role in the contractile response of the urinary bladder in many species. CCh-induced detrusor contractions are mainly mediated by muscarinic receptors M3 and depend on Ca^2+^ release from intracellular stores and influx via L-type Ca^2+^ channels. The main part of detrusor muscle contraction in an overactive human bladder is thought to be mediated via stimulation of muscarinic receptors^11^.

To date, several studies have described the effect of CCh exposure on detrusor tissue. In 2002, Wuest *et al*. evaluated the response of detrusor muscle of different species (pig, guinea pig, and a mouse) to pharmaceutical substances used in the overactive bladder treatment. They exposed detrusor muscle strips of different species to increasing concentrations of CCh (from 0.01 to 100 µM in guinea pigs and pigs, and 0.1 to 100 µM in mice) with an exposure time of 15 minutes and 2–10 minute washout between two additions of CCh. Tissue strips response was measured as a percentage of the maximum force induced by CCh. Force development initially increased to a transient peak (phasic response) and then declined to a new plateau level (tonic response). This was superimposed by spontaneous force oscillations of varying intensity. The CCh concentration-response curve reported was relatively steep^7^. Comparable responses were observed by Elliot *et al*. in 1992. When exposing rat detrusor muscle strips to increasing concentrations of CCh, the dose-response curve measuring the increase in tissue tension was of a similar shape, with highest increase in tension obtained between 1 and 10 µM CCh^9^. In human tissue, the dose-response curve to CCh has a similar steep shape with the highest increase in contraction observed at 0.1 to 1 µM CCh^10,12^.

Although a direct link between calcium dynamics and force exhibited by SMCs was demonstrated^28^, to date no study systematically addressed the concentration dependence of the calcium dynamics in individual detrusor SMCs. Using acute tissue dissection approach in combination with confocal imaging, we provided a viable detrusor SMC preparation that yielded an unprecedented spatial and temporal resolution of SMC calcium responses after CCh stimulation. During stimulation with increasing concentrations of CCh, individual detrusor SMCs responded physiologically with an increase in the basal intracellular calcium concentration with superimposed oscillations, which are described in the literature as being more prominent in small mammals than in human or pig detrusor tissue^7^. We identified three types of phenotypes with respect to the calcium dynamics: (i) spontaneous activity lacking a clear response to CCh, or a clear response to CCh with (ii) or without (iii) spontaneous activity prior to stimulation with CCh. Individual SMC responses were relatively heterogeneous and the superimposed spiking activity was not correlated among individual cells. CCh progressively activated SMCs within a narrow concentration range. At 25 µM CCh, all SMCs were active, and the peak of cell activity was observed between 25 and 50 µM CCh. Moreover, a visible tissue contraction occurred at already submaximal CCh concentrations, in accordance with previous force measurements^7^. Although a decline in activity was observed in some SMCs after stimulation with highest concentrations of CCh, we do not consider this phenomenon to be method-related, as similar was already observed in force-measurement studies, where there was a decline in tissue contraction force at very high concentrations^7^. We believe that these findings represent an important foundation for future research, as the effect of different pharmacological agents on calcium activity in detrusor SMCs can conceivably be investigated using this approach.

There are some differences in detrusor muscle responses of different species and between subjects of different age groups of the same species. For example, slight differences in potency of CCh-induced responses of SMCs were described between pig, guinea pig, and murine bladders by Wuest *et al*.^7^. Moreover, different types of spontaneous activity have been observed in neonatal, juvenile, and adult rats. Strips from adult rats exhibited a small amplitude, high frequency activity, whether strips from neonatal rats exhibited a large amplitude, low frequency spontaneous activity^6^. The reasons for different sensitivity to CCh stimulation remain unclear, but species-dependent differences in the muscarinic receptor density or subtypes has been proposed as a possible mechanism^7^.

The rhythmic activity of SMCs is thought to be at least partly mediated by ICC. The role of ICCs in both gastrointestinal (GI) and urinary tract is controversial^29^. While their pacemaker role is widely accepted for the GI tract, the data describing the role of ICCs in enteric neurotransmission are debatable. For the urinary tract, only a handful of studies described ICC morphology and their function in mice^26,30–34^. We were able to detect numerous ICCs on TEM micrographs in our preparation (Supplementary Fig. 1A,B) that displayed all the ultrastructural characteristics typical of ICCs. ICCs were characterized by rough endoplasmic reticulum, electron-lucent caveolae and electron-lucent intracellular vesicles. Individual ICCs tended to form contacts with neighbouring ICCs, as some cytoplasmic extensions were present between them. Further, on Fluo-4 loaded preparations, we detected numerous spindle-shaped cells that exhibited regular and repetitive calcium oscillations, and the activity of these ICC-like cells was not affected by CCh stimulation. The morphology of these Fluo-4 loaded cells was comparable to previous ICC descriptions^31,33–38^ and their rhythmic activity closely resembled ICC activity in the guinea pig bladder^28^ and rabbit urethra^39^. For a comprehensive review on this topic, see work by McCloskey from 2013^40^. Importantly, Hashitani *et al*. failed to find evidence for a pacemaking role of ICCs in the bladder of the guinea pig, as the ICC activity occurred independently of SMC activity^28^. Similarly, Gray *et al*. discovered that despite some subpopulations of ICCs in guinea pig urinary bladder are functionally innervated, spontaneous activity of ICCs was not correlated with that of neighbouring SMCs, although neurogenic stimulation remarkably synchronized the activity of both ICC and SMCs^41^. In this vein, also in our preparation we found no correlation between calcium activity of ICC-like cells and SMCs (Supplemental Fig. 1). Most probably, at least in mice, the spontaneous contractions are initiated within the SMCs and the role of ICCs is restricted to signal modulation^28,42^.

Besides defining the calcium response of detrusor SMCs to CCh stimulation, the main advantage and novelty of our method is the shorter preparation time and lack of a long equilibration period. By shortening the equilibration time to 15–20 minutes compared to up to two hours as described in the literature, the time from the beginning of the dissection to calcium imaging was as short as 90 minutes. On the other hand, there are some limitations of the method that are applicable to all bladder strip methods and have already been emphasized in other studies. For example, by dissecting the bladder, the tissue is acutely decentralized, which can cause injury and ischemia-related response, which needs to be taken into account. Next, viability of the tissue strips is limited to a few hours. Using our approach, SMCs remained viable exhibiting physiological calcium response and contraction up to four to five hours after preparation. Moreover, tissue preparation needs to be carefully performed to ensure its viability. Caution should be made with use of DMSO or ethanol as solvents. DMSO in concentrations higher than 0.1% can induce neurally-evoked contractions, while ethanol reduces the spontaneous smooth muscle contractions^6^. If this method is to be used in investigation of the effect of pharmacological agents on detrusor muscle tissue, several factors need to be taken into account when applying these substances, such as time for a drug to exhibit its effect, duration and mechanism of its action, and tissue thickness^6^.

In conclusion, our acute tissue dissection approach in combination with calcium imaging is relatively quick and easy to perform. Responses obtained after stimulation with CCh were within physiologic range and comparable to results of other studies in this field. On the other hand, very short equilibration period gives this method an advantage of a shorter preparation time. Although the main principle of bladder dissection and tissue preparation in our study follows similar principles as in other detrusor strip methods, our approach offers the complementary possibility to investigate individual SMCs within the detrusor tissue, as it offers the spatial and temporal resolution needed to study calcium responses of many individual SMCs simultaneously. In this way, it does not replace other detrusor strip methods, but rather adds an important insight into the calcium dynamics of detrusor SMCs with a single-cell resolution. One of the greatest advantages of this approach is the possibility to obtain large number of recordings, which subsequently increases the sensitivity of the experiment. From a methodological point of view, with appropriate adaptations our line of work could be extended to SMC preparations from some other organs^43^, detrusor tissue from other species^1^, and to more complex preparations including the urothelium, the muscularis mucosae, as well as the subepithelial sensory fibres and vessels, and used to study the differential roles of the muscular layers and paracrine interactions between neighbouring tissue types as well as relationship between SMCs and ICCs^44–46^. Importantly, genetically encoded calcium indicators could be used in future studies in combination with our approach instead of chemical indicators^47^. Similarly to some other tissues, in combination with multiphoton laser-scanning microscopy these indicators may enable *in vivo* imaging of bladder physiology in a partly exteriorized organ or through an optical window^48,49^. Further, from a (patho)physiological point of view, our preparation can be used in future studies of bladder overactivity^50,51^, the normal and pathological stimulation-contraction coupling and the role of various stimuli, ion channels, and intracellular secondary messengers in it^45,52–58^. Finally, various active substances and nonpharmacological approaches to overactive bladder treatment can conceivably be tested for their effects on calcium signals *in situ*^59,60^.

## Supplementary information

Supplmentary Video S1

Supplementary information
