# Supplementary material for: A Novel in situ Approach to Studying Detrusor Smooth Muscle Cells in Mice
Source: Sci Rep. 2020 Feb 14;10:2685. doi: 10.1038/s41598-020-59337-0 (PMC7021722; doi:10.1038/s41598-020-59337-0)
Supplement: Supplementary file 2 — Supplementary information [file 41598_2020_59337_MOESM2_ESM.pdf]

Supplementary Information

A Novel *in situ* Approach to Studying Detrusor Smooth Muscle Cells in Mice

Tamara Serdinšek[1], Saša Lipovšek[2,3,4,5], Gerd Leitinger[5], Igor But[1], Andraž Stožer[2,*], Jurij Dolenšek[2,3,*]

[1]Department of General Gynaecology and Urogynaecology, Clinic for Gynaecology and Perinatology, University Medical Centre Maribor, Ljubljanska 5, 2000 Maribor, Slovenia

[2]Faculty of Medicine, University of Maribor, Taborska ulica 8, 2000 Maribor, Slovenia

[3]Faculty of Natural Sciences and Mathematics, University of Maribor, Koroška cesta 160, 2000 Maribor, Slovenia

[4]Faculty of Chemistry and Chemical Engineering, Smetanova ulica 17, University of Maribor, 2000 Maribor, Slovenia

[5]Gottfried Schatz Research Center, Division of Cell Biology, Histology and Embryology, Medical University of Graz, Neue Stiftingtalstrasse 6, 8010 Graz, Austria

Corresponding authors:

Andraž Stožer
Faculty of Medicine
University of Maribor
Taborska ulica 8
2000 Maribor
Slovenia
e-mail: andraz.stozer@um.si

Jurij Dolenšek
Faculty of Medicine / Faculty of Natural Sciences and Mathematics
University of Maribor
Taborska ulica 8 / Koroška cesta 160
2000 Maribor
Slovenia
e-mail: jurij.dolensek@um.si

Supplementary Video S1

Supplementary Video S1: Calcium dynamics of SMCs during CCh stimulation. Ca^{2+}-dependent fluorescence signal are recorded from SMCs during stimulation with 25μ M CCh. A total of 200 frames recorded over a period of 200 seconds are presented at a rate of 5 frames s−1. Resolution is 512 x 512 pixels. Individual frames are pseudocoloured using fire LUT in Fiji, and black and white represent low and high intensity signals, respectively.

Supplementary Figure S1

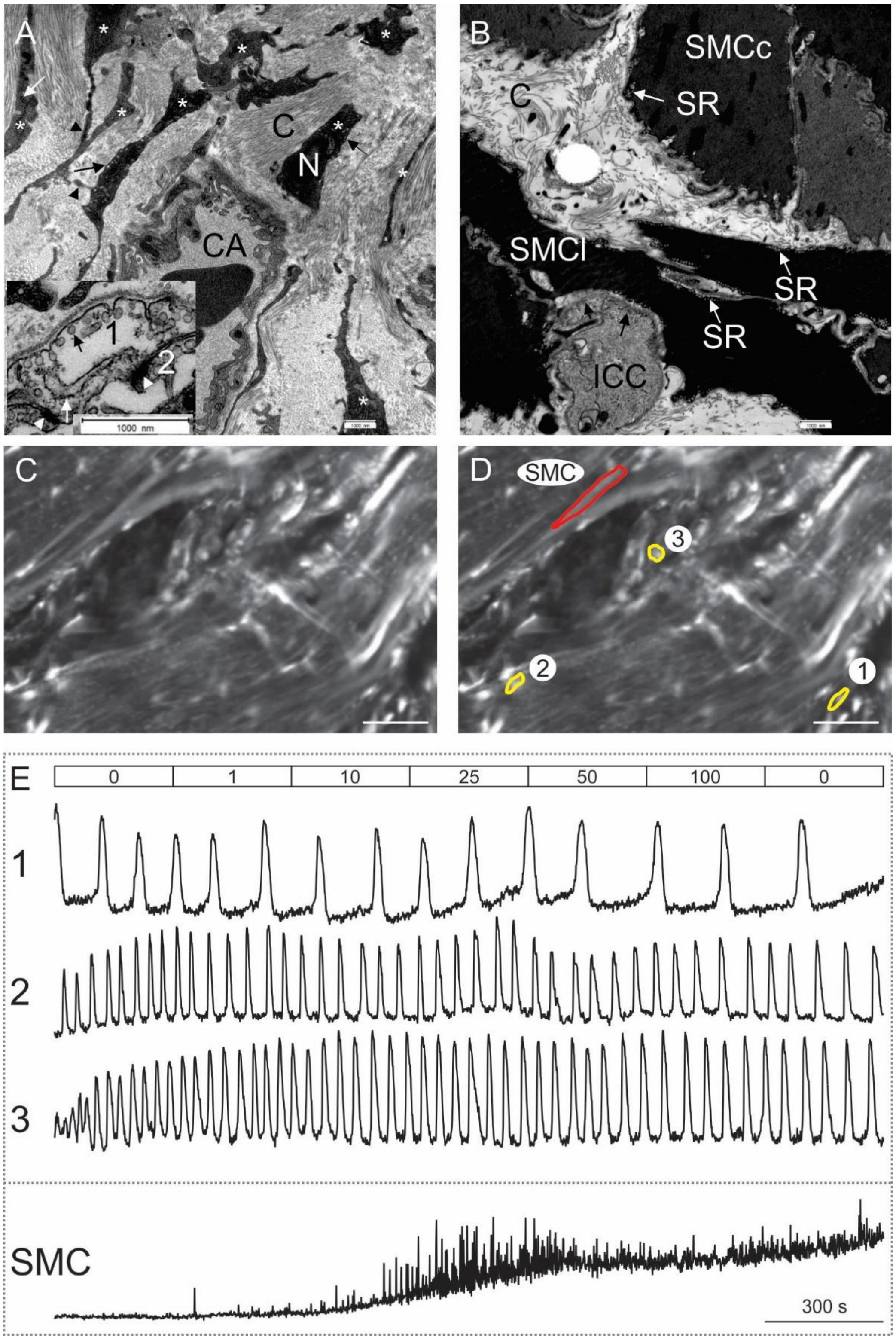

Supplementary Figure S1: Interstitial cells of Cajal (ICC). (A-B) Transmission electron micrographs demonstrating presence of ICCs. In A, we can observe ICCs (white asterisks), which are surrounded by collagen (C). Rough endoplasmic reticulum is indicated by white arrows and some electron-lucent caveolae by black arrows. Individual ICCs tend to form contacts with neighbouring ICCs, as some cytoplasmic extensions are present between them (black arrowhead). Legend: CA - capillary; N - nucleus of ICC. In the inset, two ICCs are seen. In one ICC (1), electron-lucent caveolae are shown (black arrow). Rough endoplasmic reticulum is indicated by a white arrow. Between neighbouring ICCs (1 and 2) electron dense contacts are present (white arrowheads). In B, we can observe SMCs, some of which are visible as cross-sectioned cells (SMCc), while others as longitudinally-sectioned cells (SMCl). ICC is surrounded by SMCl. In ICC, electron-lucent caveolae and vesicles are indicated by black arrow. Legend: C – collagen; SR – sarcoplasmic reticulum. (C-D) ICC-like cells were detected on Fluo-4 loaded preparations due to their small size compared to SMCs, spindle-like shape, and their position between SMCs (C). SMCs and ICC-like cells were outlined with red and yellow colour, respectively (D). (E) Calcium activity of three ICC-like cells before and during CCh stimulation, indicated by numbers that correspond to their location in panel D. For comparison, a single SMC response is also shown for the same tissue preparation (labelled with SMC and respective location indicated in panel D).

Supplementary Figure S2

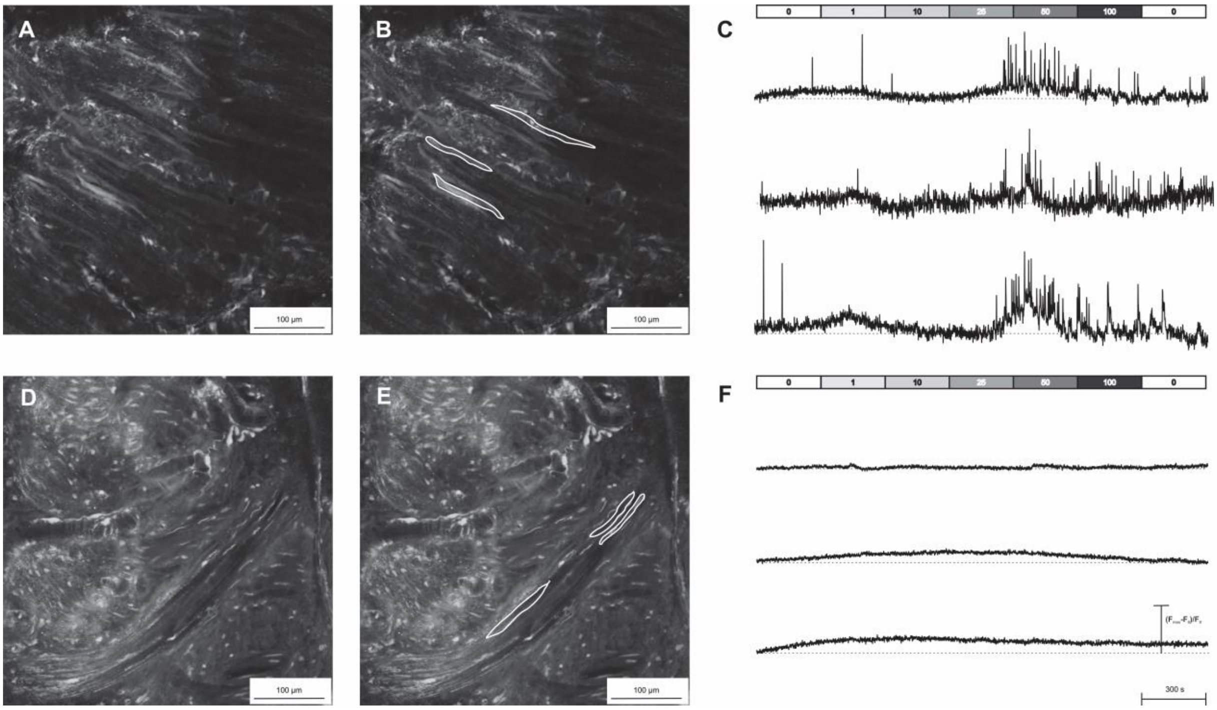

Supplementary Figure S2: Testing of two additional calcium dyes. (A) Detrusor muscle tissue loaded with OGB-1-AM. (B) Same preparation as in A, depicting the outlines of individual SMCs. (C) Calcium activity in SMCs to CCh stimulation in OGB-1-AM stained tissue. (D) Detrusor muscle tissue loaded with Rhod-2-AM. (E) Same preparation as in D, depicting the outlines of individual SMCs. (F) No calcium activity to CCh stimulation was detected in SMCs in Rhod-2-AM stained tissue.

Supplementary Figure S3

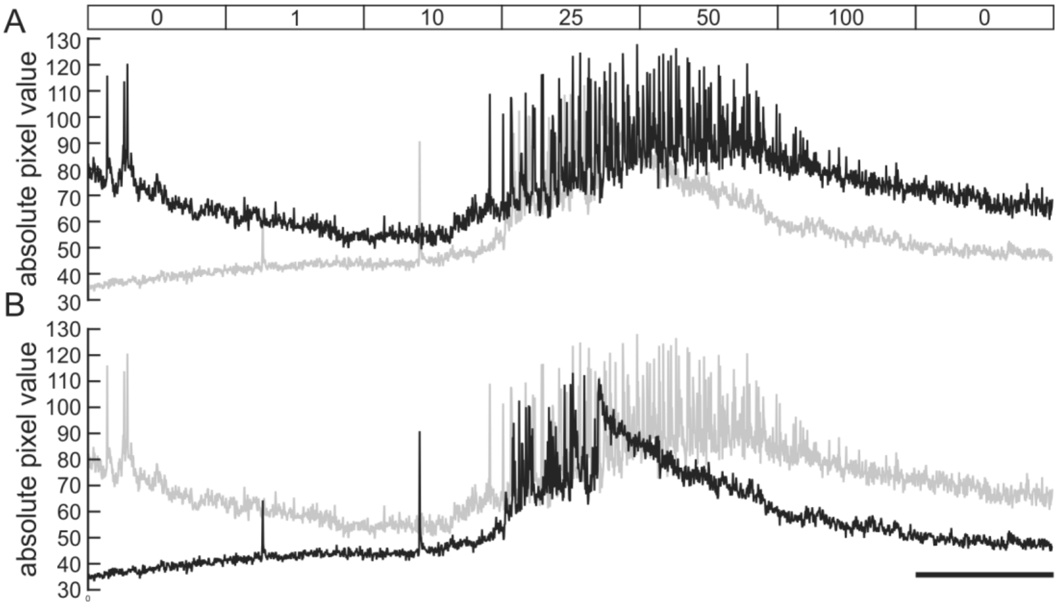

Supplementary Figure S3: The level of Fluo-4 loading did not affect SMC responses. (A) Within a single preparation, we picked the most loaded (black line) and the least loaded cell (grey line) to assess whether the level of loading affected SMC response profiles. Comparing such recordings, we observed that independently of the level of loading cells showed either spontaneous activity or the lack of it prior to CCh-induced activity, in which the responses consisted of spikes that were superimposed on the elevated plateau, and that no systematical differences between the response thresholds were observed. (B) The same pair of cells as in panel A are displayed, with colours reversed for better visualization. Scale bar indicates 300 s.
